# Increasing cell permeability of N-acetylglucosamine via 6-acetylation enhances capacity to suppress T-helper 1 (T_H_1)/T_H_17 responses and autoimmunity

**DOI:** 10.1371/journal.pone.0214253

**Published:** 2019-03-26

**Authors:** Sung-Uk Lee, Carey F. Li, Christie-Lynn Mortales, Judy Pawling, James W. Dennis, Ani Grigorian, Michael Demetriou

**Affiliations:** 1 Department of Neurology, University of California, Irvine, Irvine, California, United States of America; 2 Glixis Therapeutics, LLC, Santa Rosa, California, United States of America; 3 Department of Microbiology & Molecular Genetics, University of California, Irvine, Irvine, California, United States of America; 4 Lunenfeld-Tanenbaum Research Institute, Mount Sinai Hospital, Toronto, Ontario, Canada; 5 Department of Molecular Genetics, University of Toronto, Toronto, Ontario, Canada; 6 Department of Laboratory Medicine and Pathobiology, University of Toronto, Toronto, Ontario, Canada; 7 Institute for Immunology, University of California, Irvine, Irvine, California, United States of America; University of Lisbon, PORTUGAL

## Abstract

N-acetylglucosamine (GlcNAc) branching of Asn (N)–linked glycans inhibits pro-inflammatory T cell responses and models of autoimmune diseases such as Multiple Sclerosis (MS). Metabolism controls N-glycan branching in T cells by regulating *de novo* hexosamine pathway biosynthesis of UDP-GlcNAc, the donor substrate for the Golgi branching enzymes. Activated T cells switch metabolism from oxidative phosphorylation to aerobic glycolysis and glutaminolysis. This reduces flux of glucose and glutamine into the hexosamine pathway, thereby inhibiting *de novo* UDP-GlcNAc synthesis and N-glycan branching. Salvage of GlcNAc into the hexosamine pathway overcomes this metabolic suppression to restore UDP-GlcNAc synthesis and N-glycan branching, thereby promoting anti-inflammatory T regulatory (Treg) over pro-inflammatory T helper (T_H_) 17 and T_H_1 differentiation to suppress autoimmunity. However, GlcNAc activity is limited by the lack of a cell surface transporter and requires high doses to enter cells via macropinocytosis. Here we report that GlcNAc-6-acetate is a superior pro-drug form of GlcNAc. Acetylation of amino-sugars improves cell membrane permeability, with subsequent de-acetylation by cytoplasmic esterases allowing salvage into the hexosamine pathway. Per- and bi-acetylation of GlcNAc led to toxicity in T cells, whereas mono-acetylation at only the 6 > 3 position raised N-glycan branching greater than GlcNAc without inducing significant toxicity. GlcNAc-6-acetate inhibited T cell activation/proliferation, T_H_1/T_H_17 responses and disease progression in Experimental Autoimmune Encephalomyelitis (EAE), a mouse model of MS. Thus, GlcNAc-6-Acetate may provide an improved therapeutic approach to raise N-glycan branching, inhibit pro-inflammatory T cell responses and treat autoimmune diseases such as MS.

## Introduction

Cell surface and secreted proteins are co- and post-translationally modified on Asn (*N*) by the addition of carbohydrates (*N*-glycans) in the endoplasmic reticulum. Remodeling in the Golgi leads to hybrid and complex N-glycans that harbor one to four N-acetylglucosamine branches. Subsequent extension with β1,4 linked galactose results in N-acetyllactosamine (LacNAc), the primary ligand for the galectin family of sugar-binding proteins. Galectins bind to N-glycans in proportion to the number of LacNAc units present in hybrid and complex N-glycans [1,2]. At the cell surface, galectin–glycoprotein interactions form a macro-molecular lattice that simultaneously controls the movement, clustering, signaling and/or endocytosis of multiple receptors and transporters in a coordinated fashion to control cell growth, differentiation and death [1,3–7]. In T cells, N-glycan branching and the galectin–glycoprotein lattice inhibit T cell receptor (TCR) clustering and signaling at the immune synapse [3,8], promote central tolerance during T cell development [9], enhance CTLA-4 mediated growth arrest by inhibiting surface loss to endocytosis [1] and block pro-inflammatory T helper (T_H_) T_H_17 and T_H_1 differentiation while promoting anti-inflammatory regulatory T cell (T_reg_) responses [10,11]. N-glycan branching drives T_reg_ differentiation by promoting IL-2Rα (CD25) surface expression and signaling in T cell blasts, with loss of branching virtually eliminating induction of T_regs_ [10]. Consistent with these phenotypes, reductions in *N*-glycan branching promote pro-inflammatory T cell responses and autoimmunity in both mice and humans [3,12–17].

**Fig 1:**
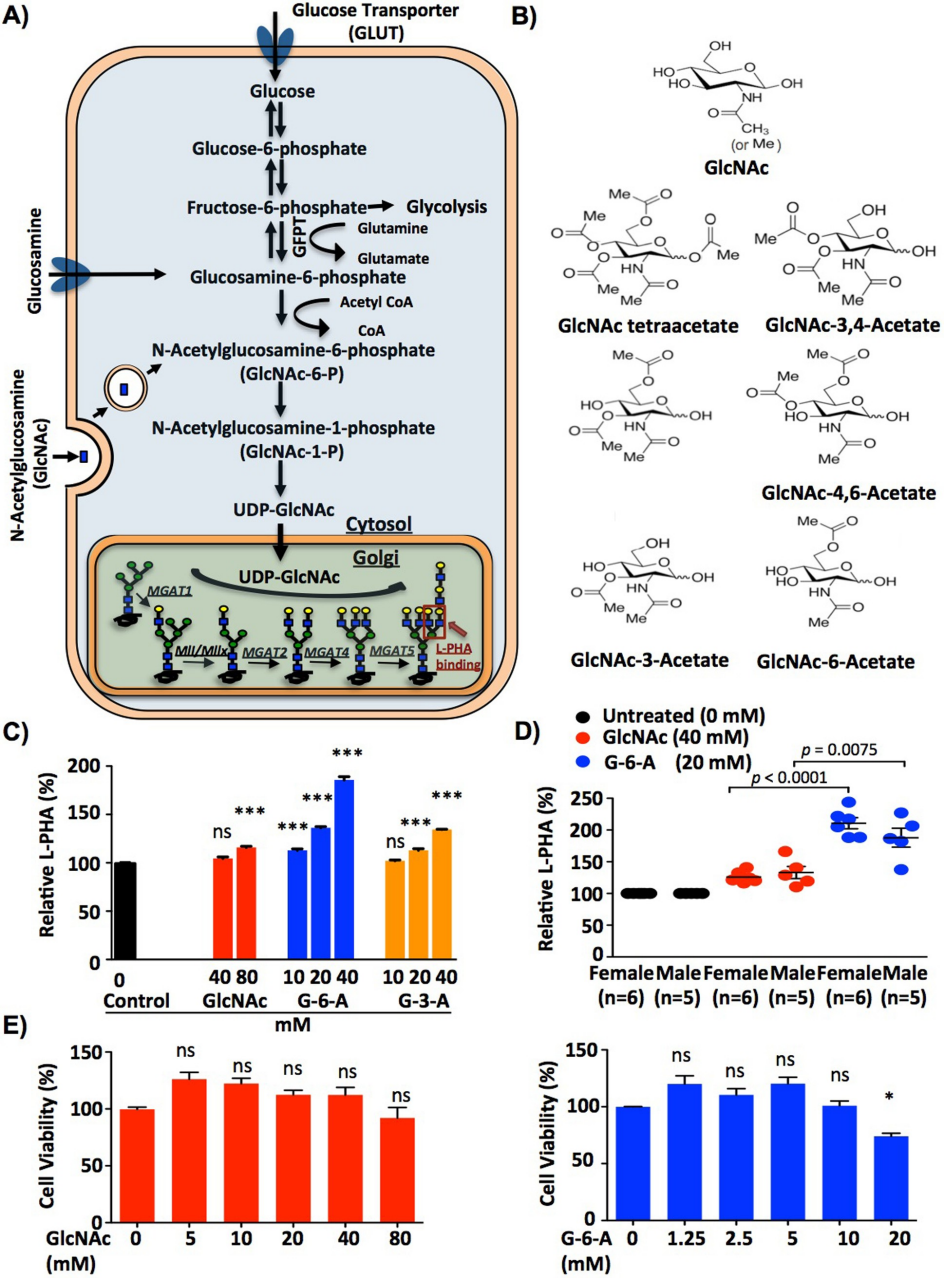
GlcNAc-6-Acetate increases N-glycan branching in both human and mouse T cells *in vitro*. (A) Complex N-glycan biosynthesis by the hexosamine and N-glycan pathways including key metabolites is shown. (B) Chemical structures of GlcNAc and different acetylated forms assessed are described. (C) Human CD4^+^ T cells were cultured with GlcNAc, GlcNAc-6-Acetate **(**G-6-A) or GlcNAc-3-Acetate **(**G-3-A) as indicated and stimulated with PMA (5 ng/ml) plus ionomycin (125 ng/ml). Cells were collected on day 5 and analyzed by flow cytometry for L-PHA staining. The bar graph and histograms shown are representative of three independent experiments. Error bars represent the mean ± standard error of triplicate treatments. (D) Treating mouse *ex vivo* splenocytes with GlcNAc-6-Acetate **(**G-6-A) raised N-glycan levels in T cells in both male and female mice. Relative L-PHA (%) was normalized to media only control. Each symbol represents one mouse. (E) Viability of human CD4^+^ T cells were measured by MTT assay after activation with anti-CD3ε plus anti-CD28 with different concentrations of GlcNAc or GlcNAc-6-Acetate. The experiments were repeated at least three independent times with similar results. *p* values in Fig 1C and 1E were determined by one-tailed ANOVA and Bonferroni’s multiple comparison test and *p* values in Fig 1D were determined by one-tailed t-test. As indicated, * *p<*0.05, ** *p<*0.01 and *** *p<*0.001.

A critical regulator of N-glycan branching is metabolism [1,10,18]. The branching *N*-acetylglucosaminyltransferases encoded by the *Mgat* gene family all utilize UDP-GlcNAc as the donor substrate; however, they do so with declining efficiency such that metabolic production of UDP-GlcNAc is limiting for Mgat4 and 5 activity (**Fig 1A**)[1]. In this manner, metabolic changes in the biosynthesis of UDP-GlcNAc by the hexosamine pathway can have marked effects on N-glycan branching. *De novo* synthesis of UDP-GlcNAc requires both glucose and glutamine, the latter as an amine donor for conversion of fructose-6-phosphate to glucosamine-6-phosphate. Rapidly dividing cells like activated T cells undergo profound metabolic changes that alter glucose and glutamine metabolism. Blasting T cells switch from the complete oxidation of glucose via oxidative phosphorylation to aerobic glycolysis and glutaminolysis, where glucose is fermented to lactate despite the presence of oxygen and glutamine is converted to α-ketoglutarate to enter the Krebs cycle [19–21]. This markedly reduces flux of glucose and glutamine into the hexosamine pathway, thereby limiting *de novo* UDP-GlcNAc biosynthesis and N-glycan branching to drive T cell growth and pro-inflammatory T_H_17 over anti-inflammatory iT_reg_ differentiation [10]. In this manner, the metabolic switch from oxidative phosphorylation to aerobic glycolysis and glutaminolysis promotes pro-inflammatory T cell responses by stealing glucose and glutamine away from the hexosamine pathway to lower N-glycan branching.

The metabolic constraint on *de novo* UDP-GlcNAc biosynthesis imposed by aerobic glycolysis and glutaminolysis can be overcome by salvage of N-acetylglucosamine (GlcNAc) into the hexosamine pathway via 6-phosphorylation (**Fig 1A**). Indeed, supplementing mouse or human T cells *in vitro* with GlcNAc enhances N-glycan branching, suppresses TCR signaling, inhibits ligand induced T cell activation and proliferation, promotes anti-autoimmune CTLA-4 surface expression and blocks pro-autoimmune T_H_1/T_H_17 differentiation while simultaneously promoting anti-autoimmune T_reg_ differentiation [1,10,13,18]. Remarkably, GlcNAc not only inhibits T_H_17 cell differentiation, but induces a cell fate switch to T_reg_ cells despite T_H_17 inducing conditions [10]. GlcNAc is also active *in vivo*, with oral GlcNAc supplementation raising N-glycan branching to inhibit autoimmune T_H_1/T_H_17 responses, spontaneous autoimmune diabetes in non-obese diabetic mice, inflammatory bowel disease in mice and progression of Experimental Autoimmune Encephalomyelitis (EAE), a mouse model for MS [18,22]. GlcNAc has also been given orally in pediatric treatment-resistant inflammatory bowel disease, where 8 of 12 children with severe disease went into clinical remission without reported toxicities [23]. A pilot study of low-dose oral GlcNAc (3g/day) effectively increased serum GlcNAc levels and mildly increased N-glycan branching in T cells of MS patients (M. Demetriou unpublished data).

An impediment to development of GlcNAc as a therapeutic for autoimmune diseases is the high concentrations required for cell entry. As GlcNAc lacks a cell surface transporter and enters cells via macropinocytosis, concentrations of 40–80 mM are required to raise N-glycan branching in T cells *in vitro*. Others have shown that lipophilicity and cell entry of N-acetylated amino sugars can be increased by masking hydroxyl groups with ester-linked acetates [24–26]. Once in the cytoplasm, endogenous esterases can remove the acetates to release the native sugar and allow utilization by metabolic pathways [24–26]. For example, per-acetylation is a widely used technique to markedly increase uptake of N-acetylated amino sugars, but toxicity may arise from incomplete de-acetylation in the cell [24,25,27,28]. Here we screened various per-, bi- and mono-acetylated forms of GlcNAc for improved activity and reduced cellular toxicity. The data identified GlcNAc-6-Acetate as a highly active pro-drug form of GlcNAc based on the ability to raise N-glycan branching greater than GlcNAc and suppress pro-inflammatory T cell responses and autoimmunity.

## Materials and methods

### Cell culture and reagents

Human CD4^+^ T cells purified from peripheral blood mononuclear cells (PBMCs) using EasySep^TM^ human CD4^+^ T cell enrichment kit (StemCell Technologies, Vancouver, Canada) were stimulated with plate-bound anti-CD3ε (OKT3, eBioscience, San Diego, CA) plus soluble anti-CD28 (CD28.2 eBioscience, San Diego, CA). Procedures with human subjects were approved by the Institutional Review Board of the University of California, Irvine and were conducted in conformity with the 1954 Declaration of Helsinki in its currently applicable version. Similarly, mouse T cells were stimulated with plate-bound anti-CD3ε (2C11, eBioscience, San Diego, CA) plus soluble anti-CD28 (37.51, eBioscience, San Diego, CA). *Mgat5* wild-type and heterozygous mice were used for mouse experiments and approved by the Institutional Animal Care and Use Committee of the University of California, Irvine. Both human and mouse cells were cultured in RPMI 1640 medium, supplemented with 10% fetal bovine serum, 2 mM L-glutamine, 100 units/ml penicillin, and 100 ug/ml streptomycin, and 50 μM β-mercaptoethanol. GlcNAc was obtained from Wellesley Therapeutics Inc. (Toronto, Canada) while all acetylated forms of GlcNAc were obtained from Santa Cruz Biotechnology (Dallas, TX). GlcNAc and its acetylated forms were >95% pure.

### MTT assay and Annexin V/7-AAD staining

Viability of Human CD4^+^ T cells at the different concentrations of GlcNAc-6-Acetate or GlcNAc was determined by using the MTT (3-(4,5-dimethylthiazol-2-yl)-2,5-diphenyltetrazolium bromide) assay (Sigma) and Annexin V Apoptosis Detection Kit (eBioscience). For the MTT assay, MTT substrate was added to cells in culture and incubated for 1 to 4 hrs. Then, the quantity of formazan (directly proportional to the number of viable cells) was measured in absorbance at 570 nm by a spectrophotometer. For Annexin V staining, cells were resuspended/stained in 1X binding buffer and incubated on ice for 30 mins. 7-AAD was added before analyzing by flow cytometry.

### Flow cytometry and L-PHA staining

Cells were washed with FACS buffer (PBS containing 0.1% (w/v) sodium azide and 2% BSA) and stained with fluorescent conjugated *Phaseolus vulgaris* leukoagglutinin lectin (L-PHA, 2 μg/ml), anti-CD4 (RPA-T4), anti-CD69 (FN50), anti-CD25 (BC96), anti-CTLA-4 (14D3) and anti-IL-17A (eBio64Dec17) for human, and anti-CD4 (RM4-5), anti-CD25 (PC61.5), anti-IL-17A (eBio17B7) and anti-FoxP3 (FJK-16s) for mouse (antibodies from eBioscience, L-PHA from Vector Labs). Proliferation was assessed by staining cells with 5, 6-carboxyfluorescein diacetate succinimidyl ester (CFSE: ThermoFisher Scientific). After staining, cells were washed twice with FACS buffer and analyzed for CFSE dilution by the BD Accuri, BD FACSCanto II, or Attune Acoustic Focusing Cytometry. Data analysis was performed using FlowJo software.

### LC-MS/MS metabolomics

Human CD4^+^ T cells were purified using the EasySep^TM^ human CD4^+^ T cell enrichment kit (StemCell Technologies, Vancouver, Canada). For the time course experiment, 3 x 10^6^ T cells per well (6 well-plate) were activated with plate-bound anti-CD3e (OKT3) plus soluble anti-CD28 (CD28.2), and harvested at 12, 24 and 48 hrs. Isolated CD4^+^ T cells were flash frozen and stored in liquid nitrogen until use for Liquid Chromatography-tandem Mass Spectroscopy (LC-MS/MS). Metabolites of frozen CD4^+^ T cell pellets were extracted as previously reported [29,30]. Briefly, 200ul solution of extraction solvent (40%:40%:20% = Acetonile: Methanol: Water) were added to the frozen pellet, and then all supernatants were transferred to clean eppendorf tubes and Speedvac dried. Each pellet was reconstituted in 150 μl of water containing internal standards D7-Glucose and UL13C915N-Tyrosine, and then transferred into mass spec vials. The samples were injected twice for negative and positive MS detection modes, through HPLC (Dionex Corporation, CA) in gradient reverse phase chromatography. The LC-MS/MS methodology was followed as reported, using a more sensitive 5500 Qtrap Mass Spectrometer (AB Sciex, Toronto, ON, Canada) for measurement of the metabolites. GlcNAc-6-Acetate ions were determined using a pure standard of GlcNAc-6-Acetate and in water the data is linear from 10 μM to 156 nM. All data is from 6 biological replicates (6 wells of a 6 well plate). Output is Area Ratio/cell number and reflects an average of 6 replicates.

### Cytokine analysis

Supernatants from human CD4^+^ T cells stimulated for 5 days *in vitro* were harvested and assessed for cytokines using the 13-plex LEGENDplex human Th cytokine panel (Biolegend, San Diego, CA) according to the manufacturer's protocol. Results are shown as mean of duplicates from two independent experiments or greater values ± S.E.

### EAE induction and oral GlcNAc/GlcNAc-6-Acetate treatment

EAE was induced by subcutaneous immunization with 100 μg of MOG 35–55 peptide (AnaSpec) for C57BL/6 mice or 100 μg MBP (Sigma) for PL/J mice. Antigens were emulsified in Complete Freund's adjuvant (Sigma) containing 4mg/ml heat-inactivated *Mycobacterium tuberculosis* (H37RA; Difco). Injections were distributed over two spots on the hind flank. All mice received 400 ng of pertussis toxin (List Biological Laboratories) by intra-peritoneal injection on days 0 and 2 after immunization. Mice were examined daily for clinical signs of EAE over the next ~30 days with the observer blinded to treatment conditions. Blinded scores were as follows: 0, no disease; 1, loss of tail tone; 2, hindlimb weakness; 3, hindlimb paralysis; 4, forelimb weakness or paralysis and hindlimb paralysis; 5, moribund or dead. Mice were treated orally with GlcNAc or GlcNAc-6-Acetate by supplementing the drinking water at 0.25 mg/ml; starting 5 days prior to immunization for the prevention model or starting on the second day of clinical disease (EAE score 1) for the treatment model, and then continued for the duration of the study. Oral consumption by all mice was verified by measuring the amount of drinking water left over after each treatment. All procedures and protocols with mice were approved by the Institutional Animal Care and Use Committee of the University of California, Irvine. Statistical analysis and *p* values for EAE mean clinical scores were determined by the Mann-Whitney test.

### Statistical analysis

Statistical analyses was by one-tailed t-test or one-tailed ANOVA with Bonferroni's multiple comparison test as indicated in the figure legends, except for EAE experiments which utilized a Mann-Whitney test. Statistical testing was performed employing GraphPad Prism 5 (GraphPad Software, Inc., La Jolla, CA).

## Results

### Acetylated forms of GlcNAc increase N-glycan branching in T cells

The biological activity of GlcNAc is limited by its inefficient membrane permeability and poor cell entry. To increase hydrophobicity and membrane permeability, we examined GlcNAc analogs modified using one or more acetyl substituents at various positions (**Fig 1B**) and tested their ability to raise N-glycan branching in human CD4^+^ T cells. Cells were activated with PMA/ionomycin or plate-bound anti-CD3ε plus soluble anti-CD28 and assessed by flow cytometry with *Phaseolus vulgaris* leukoagglutinin (L-PHA), a plant lectin that specifically binds β1,6GlcNAc-branched N-glycans produced by the Mgat5 enzyme (**Fig 1A**) and serves as an overall marker of N-glycan branching [2,3,9,10,31]. Per-acetylation of the four free hydroxyl groups in acetylated-amino sugars has been widely used to improve cell entry. However, we observed that the ability of per-acetylated GlcNAc (i.e. GlcNAc-1,3,4,6-tetraacetate) at raising N-glycan branching beyond GlcNAc was limited by toxicity (**S1A and S1B Fig**). Similarly, bi-acetylated forms of GlcNAc (i.e. GlcNAc-3,4-diacetate, GlcNAc-3,6-diacetate and GlcNAc-4,6-diacetate) were also limited in their capacity to raise N-glycan branching by toxicity (**S1C and S1D Fig)**. In contrast, mono-acetylated forms significantly raised N-glycan branching, with 10, 20 and 40mM GlcNAc-6-Acetate (G-6-A) dose-dependently increasing N-glycan branching in human T cells greater than both GlcNAc-3-Acetate (G-3-A) at 10-40mM and native GlcNAc at 20-80mM (**Figs 1C and S1E, S1F and S1H**). GlcNAc-6-Acetate was also significantly more effective than GlcNAc at raising N-glycan branching in both resting and activated mouse CD4^+^ T cells irrespective of gender (**Figs 1D and S2A**). Although 40mM GlcNAc-6-Acetate displayed the greatest increase in N-glycan branching (Fig 1C), toxicity was present based on forward/side scatter for live cells using flow cytometry (**S2B Fig**). In contrast, the enhancement of N-glycan branching induced by 10mM and 20mM GlcNAc-6-Acetate was associated with no and minimal toxicity, respectively, based on forward/side scatter for live cells on flow cytometry (**S2B Fig**), a MTT viability assay (**Fig 1E)** as well as Annexin V and 7-AAD staining using flow cytometry (**S2C Fig**). Based on this, we selected GlcNAc-6-Acetate at concentrations of 20mM or less for further characterization.

### GlcNAc-6-Acetate is salvaged into the hexosamine pathway to increase UDP-GlcNAc

**Fig 2:**
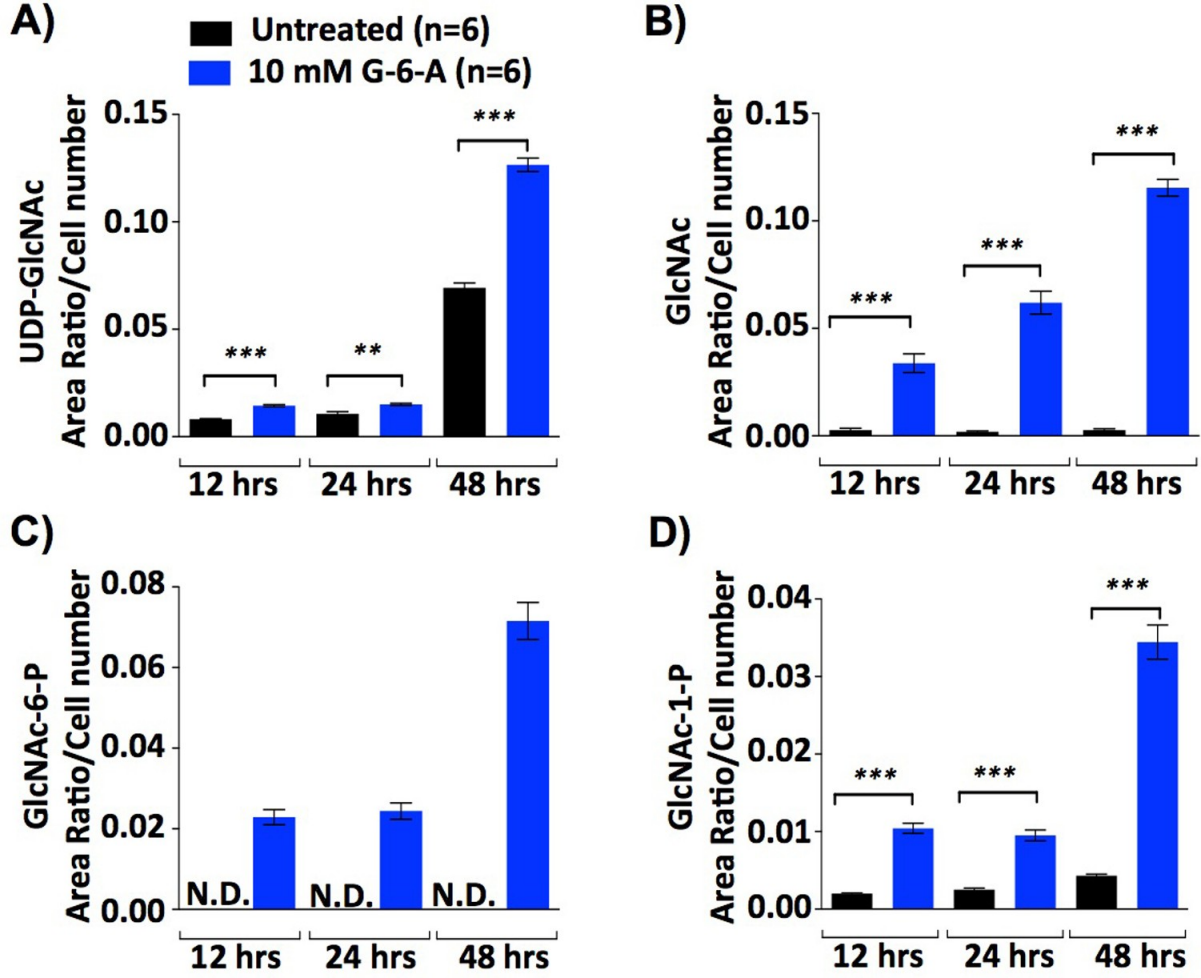
GlcNAc-6-Acetate increases metabolites in the hexosamine salvage pathway. (A-D) Human CD4^+^ T cells from a single donor were stimulated with anti-CD3ε (1 μg/ml) plus anti-CD28 (0.5 μg/ml) without (untreated) or with 10 mM GlcNAc-6-Acetate (G-6-A) and harvested at different time points (24 hr, 48 hr or 72 hrs) for mass spectroscopy analysis. Area Ratio/Cell Number of 4 key metabolites (UDP-GlcNAc, GlcNAc, GlcNAc-6-P and GlcNAc-1-P) in the pathway were determined. Area Ratio/Cell Number is the area of the metabolite peak compared to the area of the internal standard peak divided by cell number. Data are representative of two independent experiments. *p* values were determined by one-tailed t-test. Error bars represent the mean ± standard error of six replicate treatments. As indicated, * *p<*0.05, ** *p<*0.01 and *** *p<*0.001.

To confirm that GlcNAc-6-Acetate is salvaged into the hexosamine pathway and raises UDP-GlcNAc, we analyzed hexosamine pathway metabolites in lysates of activated CD4^+^ T cells by Liquid Chromatography-tandem Mass Spectroscopy (LC-MS/MS). As previously observed, T cell activation enhanced UDP-GlcNAc production over time (**Fig 2A**) [1]. Addition of 10mM GlcNAc-6-Acetate further raised UDP-GlcNAc levels along with intracellular GlcNAc, GlcNAc-6-P, and GlcNAc-1-P, indicating GlcNAc-6-Acetate was de-acetylated and salvaged into the hexosamine pathway to raise UDP-GlcNAc (**Fig 2A–2D, see Fig 1A for pathway**).

### GlcNAc-6-Acetate is superior to GlcNAc at inhibiting T cell activation and growth

**Fig 3:**
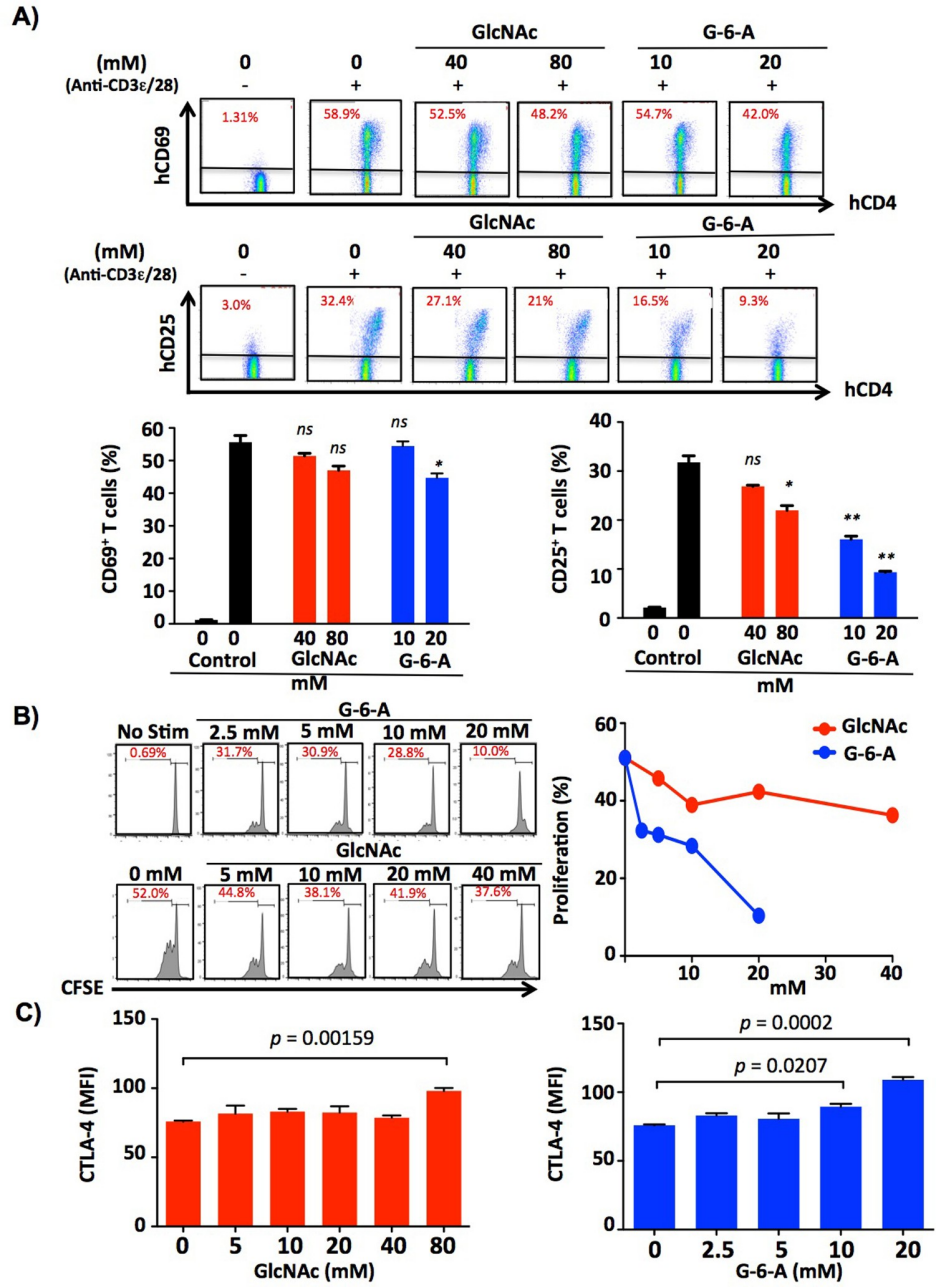
GlcNAc-6-Acetate inhibits human T cell activation *in vitro*. (A) Human CD4^+^ T cells were stimulated with anti-CD3ε (1 μg/ml) plus anti-CD28 (0.5 μg/ml) with indicated compounds and then collected at 24 hours for the early activation marker CD69 and at 72 hours for the late activation marker, CD25. *p* values were determined by one-tailed t-test and as indicated, * *p*< 0.05, ** *p* < 0.01 and *** *p*< 0.001. Data are representative of three independent experiments. Error bars represent the mean ± standard error of triplicate treatments. (B) Human CD4^+^ T cells were labeled with CFSE (final concentration = 1 uM), were activated for 5 days and then analyzed by flow cytometry. Data are representative of two independent experiments. (C) Human CD4^+^ T cells were stimulated with anti-CD3ε (1 μg/ml) plus anti-CD28 (0.5 μg/ml) for 6 days and then analyzed for CTLA-4 surface expression by flow cytometry. Data are representative of two independent experiments. *p* values were determined by one-tailed ANOVA and Bonferroni’s multiple comparison test. Error bars represent the mean ± standard error of duplicate treatments.

N-glycan branching and GlcNAc suppress T cell activation and growth by inhibiting T cell receptor clustering at the immune synapse and promoting surface retention of the growth inhibitor CTLA-4 [1,3,8,18]. To assess effects of GlcNAc-6-Acetate-mediated increases in N-glycan branching on T cell activation and growth, purified CD4^+^ T cells were activated for 3–6 days *in vitro* with/without GlcNAc at 40 and 80mM or GlcNAc-6-Acetate at 10 and 20mM. Compared to GlcNAc, GlcNAc-6-Acetate was more effective at inhibiting induction of the early T cell activation marker CD69 at 24 hours and the late T cell activation marker CD25 at 72 hours (**Fig 3A**). Similarly, GlcNAc-6-Acetate was more effective than GlcNAc at inhibiting proliferation of human (**Fig 3B**) and mouse T cells (**S3A Fig**). The increase in surface levels of the growth inhibitor CTLA-4 in human T cell blasts was also greater with GlcNAc-6-Acetate compared to GlcNAc (**Fig 3C**).

### GlcNAc-6-Acetate is superior to GlcNAc at inhibiting pro-inflammatory T cell responses *in vitro*

**Fig 4:**
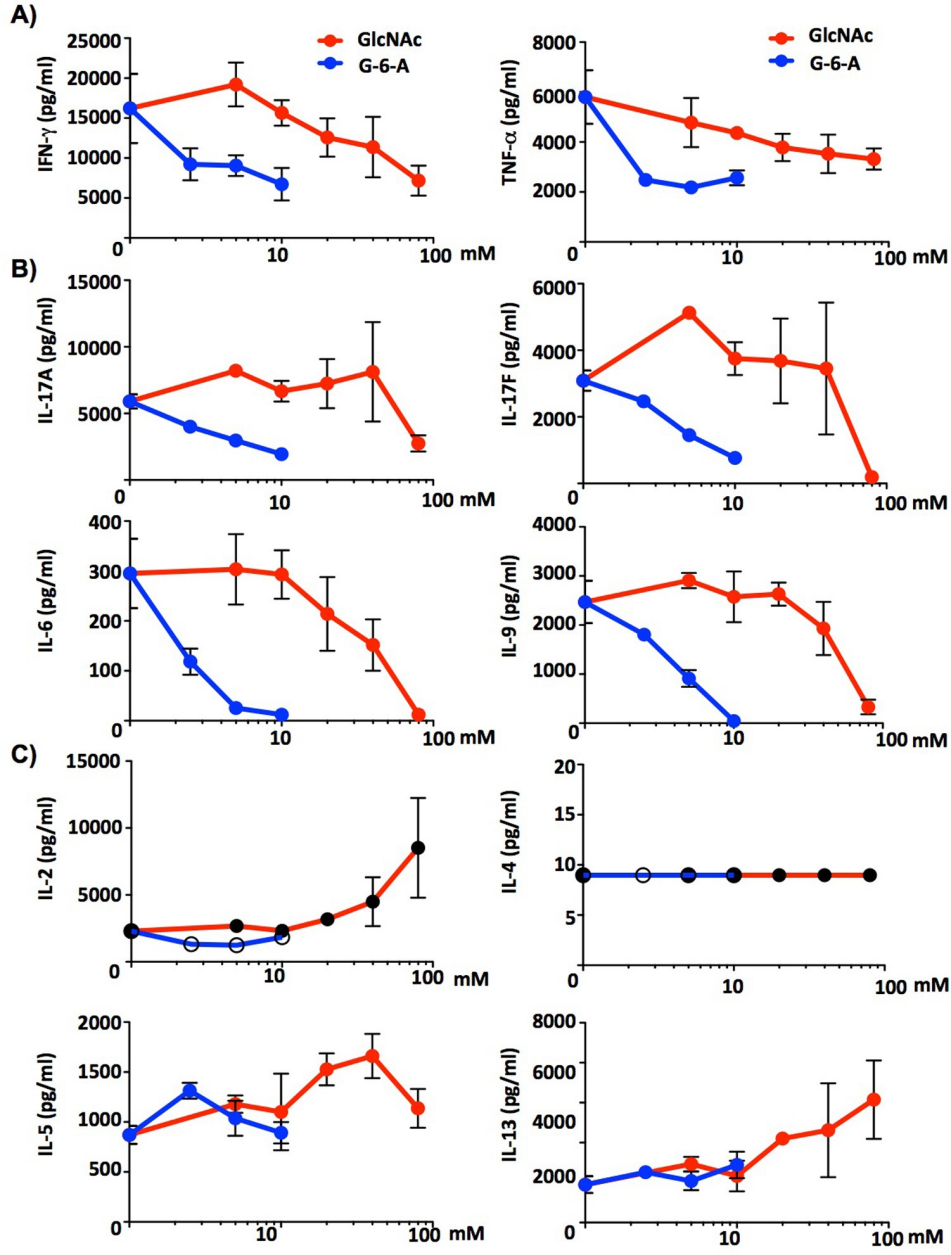
GlcNAc-6-Acetate inhibits production of T_H_1/T_H_17 cytokines. (A-C) Human CD4^+^ T cells were stimulated with anti-CD3ε (1 μg/ml) plus anti-CD28 (0.5 μg/ml) for 5 days with different concentrations of GlcNAc (0, 5, 10, 20, 40 and 80 mM) or GlcNAc-6-Acetate **(**0, 2.5, 5, 10 mM). Supernatants were analyzed with flow cytometry-based multiplex cytokine assay for T_H_1 (IFN-γ and TNF-α, T_H_17 (IL-17A, IL-17F, IL-6 and IL-9) and IL-2, T_H_2 (IL-4, IL-5, IL-13) cytokines. Data shown are representative of two independent experiments. Error bars represent standard deviation of triplicate treatments.

Activated T cells differentiate into pro- or anti-inflammatory sub-types depending on the degree of TCR activation and co-signals from cytokines. N-glycan branching and GlcNAc inhibit pro-inflammatory T_H_1 and T_H_17 differentiation while promoting anti-inflammatory T_H_2 responses. To assess the effect of GlcNAc-6-acetate on these responses, healthy human CD4^+^ T cells were activated under neutral conditions for 5 days with/without GlcNAc or GlcNAc-6-Acetate and at day 5, supernatants were harvested and analyzed by a flow cytometry-based cytokine assay (Biolegend, San Diego). As observed with T cell activation and growth, GlcNAc-6-Acetate was more effective compared to GlcNAc at inhibiting production of the pro-inflammatory T_H_1 cytokines IFN-γ and TNF-α. Similarly, GlcNAc-6-Acetate was also superior to GlcNAc at inhibiting pro-inflammatory T_H_17 cytokine secretion (IL-17A and IL-17F) as well as reducing cytokines that promote T_H_17 differentiation (IL-6 and IL-9) (**Fig 4C**). Under T_H_17 inducing conditions, GlcNAc-6-Acetate was also more effective than GlcNAc at inhibiting autoimmune T_H_17 differentiation (**S3B Fig**). In contrast, GlcNAc-6-Acetate had little impact on IL-2 and the T_H_2 cytokines IL-4, IL-5 and IL-13 (**Fig 4C**).

### Oral administration of GlcNAc-6-Acetate increases N-glycan branching to suppress T cell responses and autoimmunity in mice

**Fig 5:**
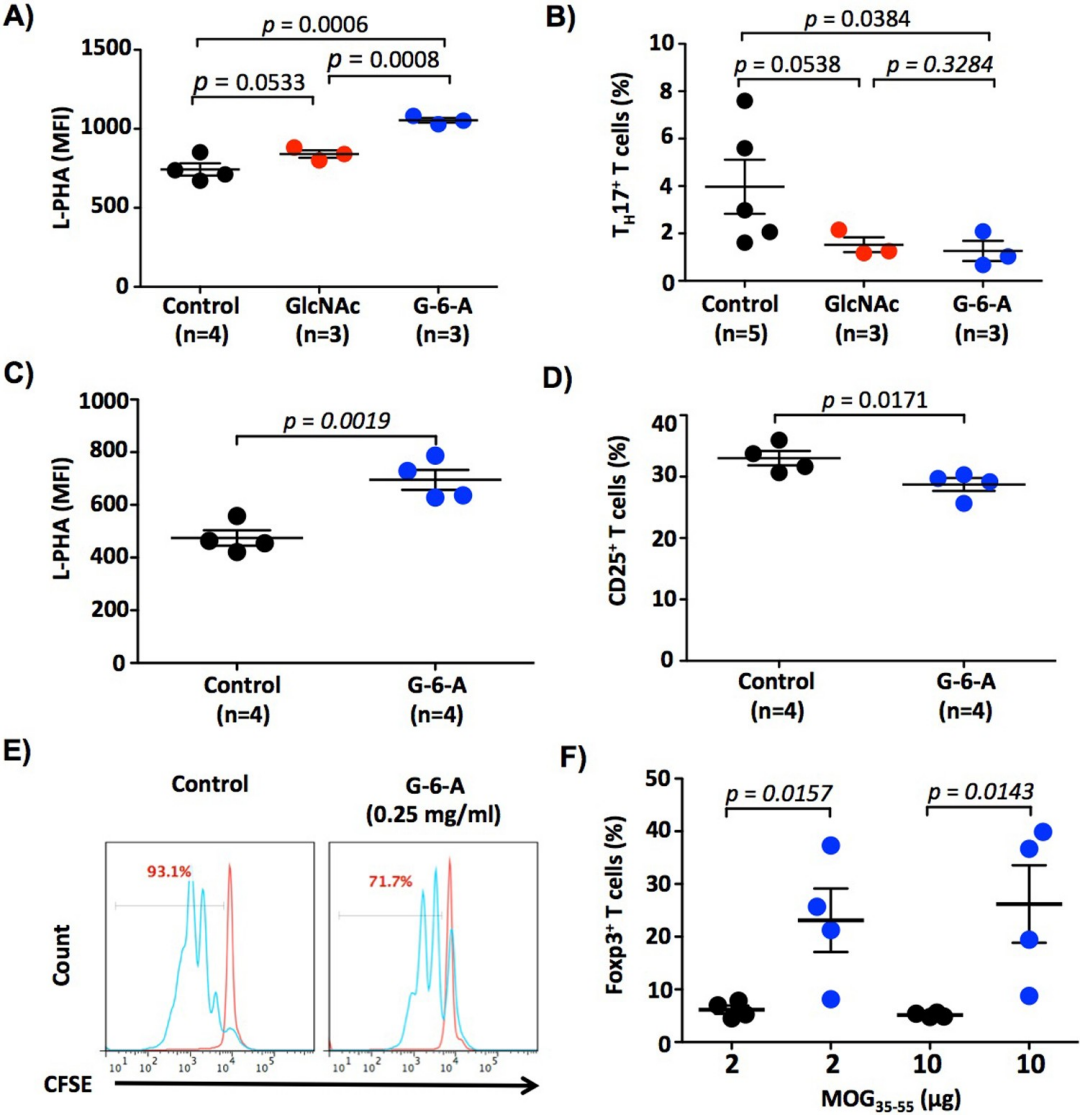
Oral administration of GlcNAc-6-Acetate increases N-glycan branching and inhibits T cell activation/growth *in vivo*. (A-F) C57BL/6 Mgat5^+/-^ mice were provided GlcNAc or GlcNAc-6-Acetate **(**G-6-A) at 0.25 mg/ml in their drinking water daily 5 days prior to MOG_35-55_ immunization. (A,B) 5 days post-immunization, inguinal lymph nodes (A) and splenocytes (B) were harvested and stained/analyzed by flow cytometry for L-PHA staining (A) or re-stimulated with MOG_35-55_ and analyzed 72 hours later by flow cytometry for T_H_17^+^ T cells (B). Each symbol represents a single mouse. Results in (B) were replicated by an ELISA for IL-17 in an independent experiment. (C-F) 28 days post-immunization, with mice continually treated daily with vehicle or GlcNAc-6-Acetate **(**G-6-A, 0.25 mg/ml) in the drinking water, inguinal lymph nodes (C,D) and splenocytes (E, F) were harvested and analyzed by flow cytometry for L-PHA (C), the late activation marker CD25, (D) proliferation by CFSE dilution following stimulation with anti-CD3ε plus anti-CD28 *in vitro* (E) or T_reg_ frequency by flow cytometry for FoxP3 expression following re-stimulation with MOG_35-55_ antigen (F). *p* values were determined by one-tailed t-test. Data shown in (C,D) were replicated in an independent experiment. Error bars represent the mean ± standard error of biological replicates.

In previous *in vivo* studies of oral administration of GlcNAc, we observed that blasting T cells were more sensitive to oral GlcNAc than resting T cells and that optimal effects were obtained by providing GlcNAc at 0.25 mg/ml in drinking water [18]. Therefore, here we treated Mgat5^+/-^ C57BL/6 mice with GlcNAc-6-Acetate or GlcNAc at 0.25 mg/ml for 5 days, followed by Myelin Oligodendrocyte Protein 35–55 peptide (MOG_35-55_) immunization and harvesting of draining inguinal lymph node T cells for flow cytometry 5 days later. There were no differences in the volume of water consumed by the mice (**S4 Fig**). Consistent with *in vitro* data, oral GlcNAc-6-Acetate was more effective than GlcNAc at raising N-glycan branching in CD4^+^ T cells *in vivo* (**Fig 5A**). Indeed, *ex vivo* re-stimulation of splenocytes with MOG_35-55_ revealed that T cells from GlcNAc-6-Acetate treated mice displayed reduced T_H_17 differentiation relative to both control and GlcNAc treated mice (**Fig 5B**). Longer-term treatment (28 days) of MOG_35-55_ immunized mice with oral GlcNAc-6-Acetate also raised N-glycan branching without overt clinical signs of toxicity in the mice (**Fig 5C**). Flow cytometry of *ex vivo* T cells from these mice revealed that GlcNAc-6-Acetate reduced activation *in vivo*, as assessed by the activation marker CD25 (**Fig 5D**). Moreover, oral GlcNAc-6-Acetate treatment reduced *ex vivo* T cell proliferation in response to anti-CD3ε plus anti-CD28 (**Fig 5E**) and promoted anti-autoimmune Treg differentiation following re-stimulation with MOG_35-55_ (**Fig 5F**).

**Fig 6:**
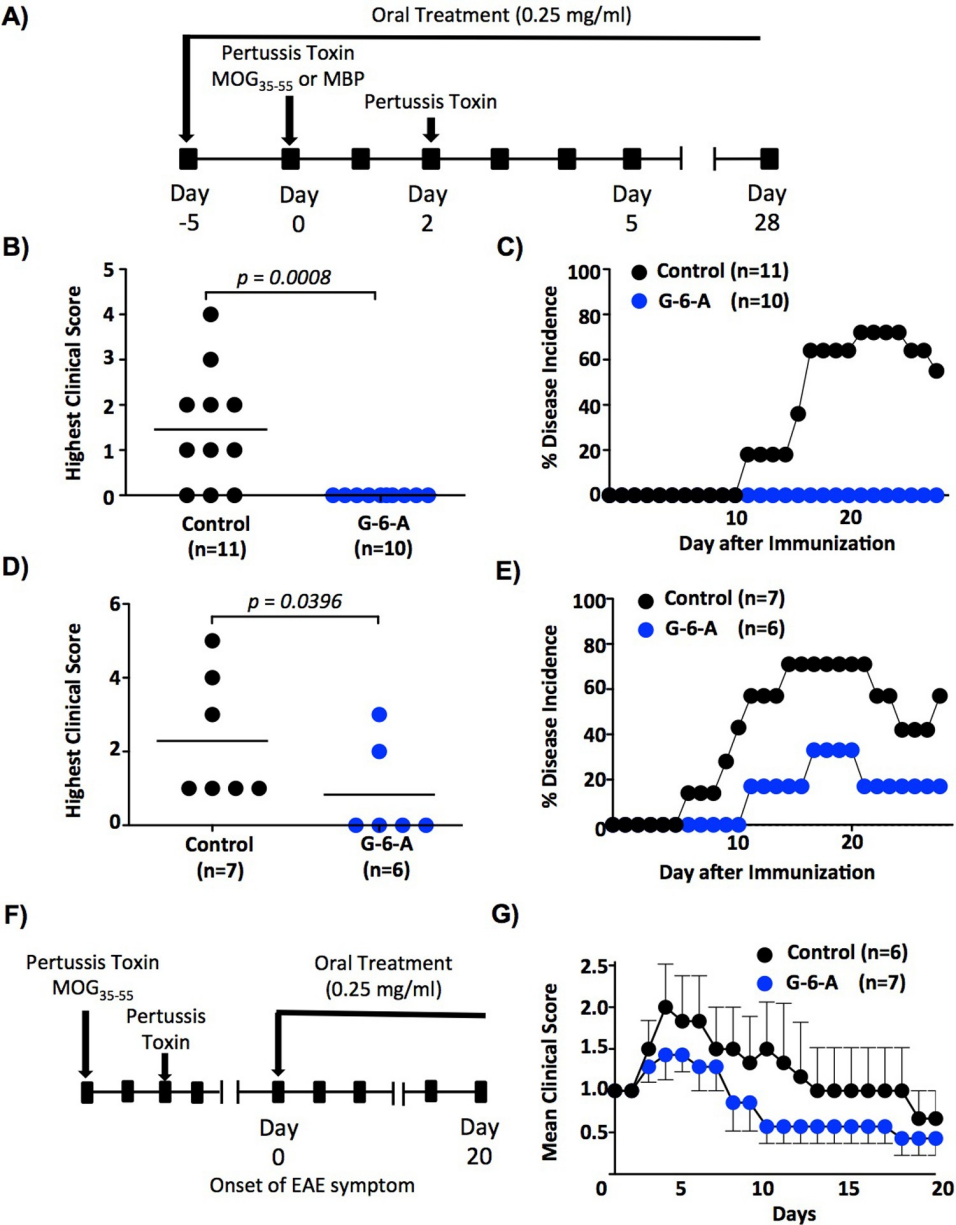
Oral administration of GlcNAc-6-Acetate suppresses EAE in both C57BL/6 and PL/J strains of mice. (A) Experimental plan for the EAE prevention study with GlcNAc-6-acetate (G-6-A). (B-E) Mgat5^+/-^ mice of the C57BL/6 strain (B-C) and the PL/J strain (D-E) were treated as in (A) and blindly monitored daily for clinical signs of EAE. Disease incidence was calculated by dividing the number of mice that displayed clinical signs of EAE (EAE score ≥1) by the total number of mice scored in the group. *p* values were determined by Mann-Whitney test. (F) Experimental plan for the EAE treatment study with GlcNAc-6-acetate (G-6-A). (G) C57BL/6 wild type mice were treated with G-6-A starting on the second day after disease onset (EAE score ≥1, Day 0) and blindly monitored daily for clinical signs of EAE. Error bars represent the mean ± standard error of biological replicates.

Next, we assessed the ability of GlcNAc-6-Acetate to inhibit clinical EAE using two different mouse models, MOG_35-55_ induced EAE model in Mgat5^+/-^ C57BL/6 mice and myelin basic protein (MBP) induced EAE model in Mgat5^+/-^ PL/J mice. Mgat5 heterozygous mice were chosen as they enhance the severity of clinical EAE yet are still sensitive to GlcNAc. Both prevention and treatment regimens were examined, with oral GlcNAc-6-Acetate treatment beginning 5 days prior to immunization for the prevention study (**Fig 6A**) or on the second day after disease onset for the treatment study (**Fig 6F**) and continued throughout both studies. Mice were assessed daily by a blinded observer for clinical signs of EAE. GlcNAc-6-Acetate significantly suppressed EAE in both C57BL/6 and PL/J EAE models when initiated prior to disease onset (**Fig 6B–6E**). When initiated after EAE onset, oral treatment with GlcNAc-6-Acetate inhibited disease progression (**Fig 6G**), where day 0 indicates the first day of clinical disease. Note that current disease modifying therapies in MS act by preventing new areas of T cell mediated demyelination, therefore EAE prevention models are the better model to assess prevention of relapses in MS patients.

## Discussion

Inefficient membrane permeability and cellular uptake limits the biological activity of GlcNAc. Efforts to increase the potency of GlcNAc directed our attention to the strategy where ester-linked acetates are employed for masking polar moieties and increasing the lipophilicity of acetylated-amino sugars, which are then unmasked via endogenous esterases for utilization by native metabolic pathways. However, the number of acetyl groups that must be removed to yield free GlcNAc may negatively impact activity and toxicity from incomplete de-acetylation inside the cell. The precise location of acetylation may also impact the rate of de-acetylation in the cell to affect function and/or toxicity [26,32]. Thus, enhancing the lipophilicity of GlcNAc via acetylation must be balanced with a requirement to maximally promote de-acetylation inside the cell by carefully controlling the number and location of the acetyl groups. Consistent with this, we observed that 6-acetylation of GlcNAc was significantly more potent at raising N-glycan branching than non-acetylated GlcNAc at concentrations displaying little or no toxicity, while activity of other mono- and bi-acetylated forms were less potent and/or limited by cell toxicity. This suggests that the extra degree of freedom of rotation at the 6-carbon position better enables access to endogenous esterase and subsequent de-acetylation inside the cell.

N-glycan branching is a critical negative regulator of T cell function and risk factor for autoimmune diseases such as MS. Metabolite flux and salvage of GlcNAc into the hexosamine biosynthetic pathway regulates T cell homeostasis and self-tolerance, offering a potential therapeutic approach to MS treatment. Indeed, we are currently conducting a Phase 1 dose-finding clinical trial of oral GlcNAc in MS patients. However, GlcNAc activity is limited by the large quantities required to enter cells, an issue that is addressed with GlcNAc-6-acetate. Indeed, consistent with superior activity at raising N-glycan branching *in vitro* and *in vivo*, GlcNAc-6-Acetate was also superior to GlcNAc at inhibiting T cell activation and pro-inflammatory differentiation. GlcNAc-6-Acetate also inhibited T cell driven inflammatory demyelination in both prevention and treatment EAE models.

There is no cure for MS, but multiple disease-modifying therapies are available. However, all current treatment strategies for MS have one or more shortcomings such as modest activity, broad immuno-suppression with risk of cancer and/or serious infections such as Progressive Multifocal Leukoencephalopathy, high toxicity, high cost, frequent injections and/or intolerable sides effects. These issues are particularly problematic as MS is a chronic disease that requires continuous treatment throughout the lifetime of the patient. Therefore, agents that have significant efficacy in the absence of broad immuno-suppression, toxicity and/or side effects would provide a significant improvement over current therapies. Our data suggests that GlcNAc-6-Acetate could serve this role, acting as a pro-drug form of a natural metabolite that is activated by intracellular esterases to increase N-glycan branching in T cells and thereby negatively regulate pro-inflammatory T cell responses in MS. GlcNAc-6-Acetate is also likely to be beneficial in many other T cell dominant autoimmune diseases, particularly inflammatory bowel disease. Detailed animal toxicity testing will be required to better assess the safety of GlcNAc-6-Acetate prior to initiating human clinical trials.

## Supporting information

S1 Fig Regulation of N-glycan branching and cell viability upon treatment with different acetylated forms of GlcNAc. (A-B) Human CD4^+^ T cells were activated for 72 hrs with different concentrations of per-acetylated GlcNAc (GlcNAc tetra-acetate (TA)) and then analyzed for L-PHA staining and cell viability using 7-AAD by flow cytometry. Bar graphs show the means ± standard error from four independent experiments. (C) Human CD4^+^ T cells or (D) CD8^+^ T cells were stimulated with PMA plus ionomycin and then analyzed by flow cytometry for L-PHA staining on 5 days with the indicated GlcNAc di-acetate analogs. Data is representative of at least two independent experiments. (E) Human CD4^+^ T cells were cultured with as indicated and stimulated with anti-CD3ε (1μg/ml) + anti-CD28 (0.5 μg/ml). Cells were collected on day 3 and analyzed by flow cytometry for L-PHA staining. The graph represents three independent experiments. Error bars represent the means ± standard error of duplicate treatments. (F) Human CD4^+^ T cells from 3 different donors with untreated or 10 mM GlcNAc-6-Acetate were stimulated with PMA plus ionomycin. Cells were collected on 5 days and stained by flow cytometry for LPHA staining. *p* values were determined by one-tailed t-test. (H) Human CD4^+^ T cells with untreated or 20 mM of GlcNAc, GlcNAc-6-Acetate or GlcNAc-3-Acetate were stimulated with PMA plus ionomycin. Cells were collected on 5 days and stained by flow cytometry for LPHA staining. The gragh was shown with the combination of two independent results. *p* values in S1E and S1H Fig were determined by one-tailed ANOVA and Bonferroni’s multiple comparison test and as indicated, * *p<*0.05, ** *p<*0.01 and *** *p<*0.001.
(TIFF)

S2 Fig GlcNAc-6-Acetate increases N-glycan branching in mouse CD4^+^ T cells over time and shows mild toxicity at 20 mM in human CD4^+^ T cells. (A) Mouse CD4^+^ T cells were stimulated without (untreated), with GlcNAc (40 mM) or G-6-A (20 mM) and analyzed at different time points. Relative L-PHA (%) was normalized to media only control. The experiment was conducted at least two independent times with similar results. (B) Human CD4^+^ T cells were activated for 5 days with different concentrations of G-6-A or GlcNAc and then analyzed by flow cytometry for cell viability based on forward/side scatter. (C) Human CD4^+^ T cells were stimulated for 3 days along with untreated, 20 mM of G-6-A or 40 mM of GlcNAc and then analyzed using Annexin V/7-AAD by flow cytometry.
(TIFF)

S3 Fig GlcNAc-6-Acetate inhibits T cells activation in mouse cells and T_H_17 differentiation in human cells *in vitro*. (A) Mouse splenic CD4^+^ T cells were labeled with CFSE, activated for 72 hrs with anti-CD3ε + anti-CD28, and treated with GlcNAc (40 mM) or G-6-A (20 mM). The experiment is representative from at least two independent experiments. (B) Human CCR6^+^CD4^+^ T cells were sorted and activated with anti-CD3ε + anti-CD28 under T_H_17-inducing conditions with control (untreated), GlcNAc (40 mM) or G-6-A (10 mM) for 5 days. The experiment is repeated twice with similar results. Error bars represent the means ± standard error of duplicate treatments. *p* values were determined by one-tailed t-test.
(TIFF)

S4 Fig Average amount of water consumed by mice supplemented with GlcNAc and GlcNAc-6-acetate in their drinking water. C57BL/6 Mgat5^+/-^ mice were provided GlcNAc or GlcNAc-6-Acetate (G-6-A) at 0.25 mg/ml in their drinking water daily 5 days prior to MOG_35-55_ immunization and for 5 days post-immunization. Shown is the average amount of water consumed per mouse per day over the 10 day period of treatment.
(TIFF)
