# Supplementary figures and images for: Increasing cell permeability of N-acetylglucosamine via 6-acetylation enhances capacity to suppress T-helper 1 (TH1)/TH17 responses and autoimmunity
Source: PLoS One. 2019 Mar 26;14(3):e0214253. doi: 10.1371/journal.pone.0214253 (PMC6435169; doi:10.1371/journal.pone.0214253)

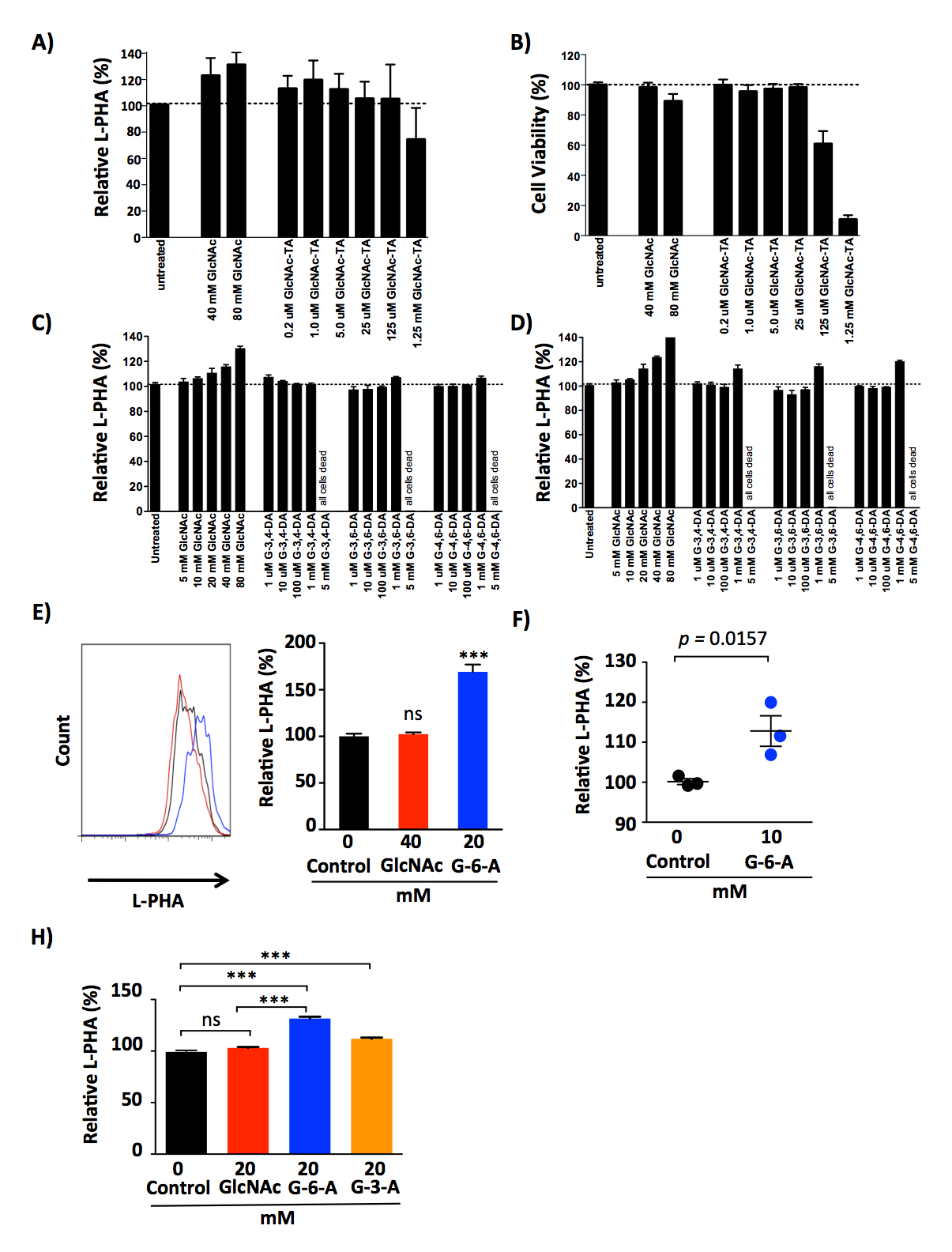

Supplement: S1 Fig — (A-B) Human CD4+ T cells were activated for 72 hrs with different concentrations of per-acetylated GlcNAc (GlcNAc tetra-acetate (TA)) and then analyzed for L-PHA staining and cell viability using 7-AAD by flow cytometry. Bar graphs show the means ± standard error from four independent experiments. (C) Human CD4+ T cells or (D) CD8+ T cells were stimulated with PMA plus ionomycin and then analyzed by flow cytometry for L-PHA staining on 5 days with the indicated GlcNAc di-acetate analogs. Data is representative of at least two independent experiments. (E) Human CD4+ T cells were cultured with as indicated and stimulated with anti-CD3ε (1μg/ml) + anti-CD28 (0.5 μg/ml). Cells were collected on day 3 and analyzed by flow cytometry for L-PHA staining. The graph represents three independent experiments. Error bars represent the means ± standard error of duplicate treatments. (F) Human CD4+ T cells from 3 different donors with untreated or 10 mM GlcNAc-6-Acetate were stimulated with PMA plus ionomycin. Cells were collected on 5 days and stained by flow cytometry for LPHA staining. p values were determined by one-tailed t-test. (H) Human CD4+ T cells with untreated or 20 mM of GlcNAc, GlcNAc-6-Acetate or GlcNAc-3-Acetate were stimulated with PMA plus ionomycin. Cells were collected on 5 days and stained by flow cytometry for LPHA staining. The gragh was shown with the combination of two independent results. p values in S1E and S1H Fig were determined by one-tailed ANOVA and Bonferroni’s multiple comparison test and as indicated, * p<0.05, ** p<0.01 and *** p<0.001. (TIFF) [file pone.0214253.s001.tiff]

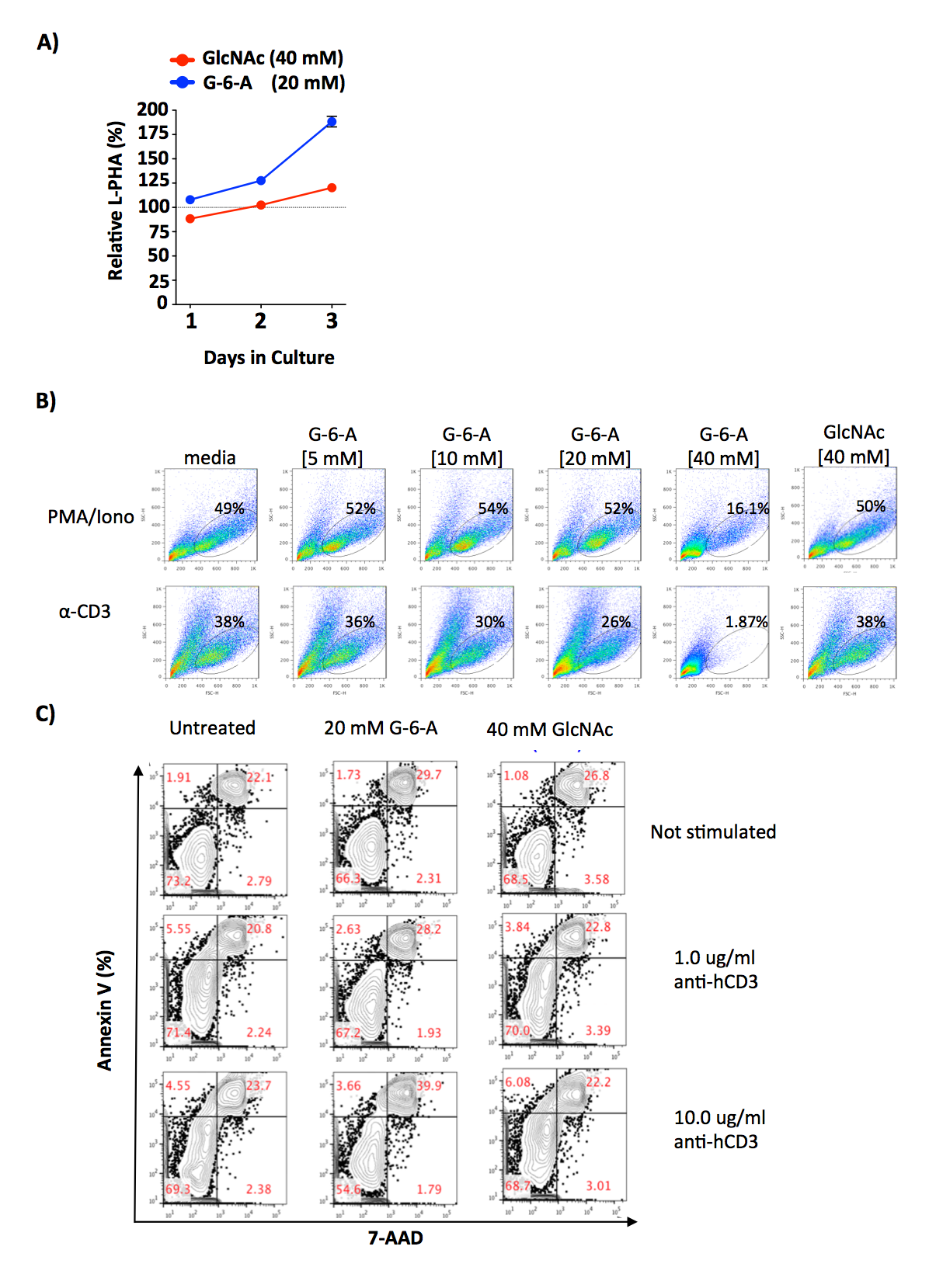

Supplement: S2 Fig — (A) Mouse CD4+ T cells were stimulated without (untreated), with GlcNAc (40 mM) or G-6-A (20 mM) and analyzed at different time points. Relative L-PHA (%) was normalized to media only control. The experiment was conducted at least two independent times with similar results. (B) Human CD4+ T cells were activated for 5 days with different concentrations of G-6-A or GlcNAc and then analyzed by flow cytometry for cell viability based on forward/side scatter. (C) Human CD4+ T cells were stimulated for 3 days along with untreated, 20 mM of G-6-A or 40 mM of GlcNAc and then analyzed using Annexin V/7-AAD by flow cytometry. (TIFF) [file pone.0214253.s002.tiff]

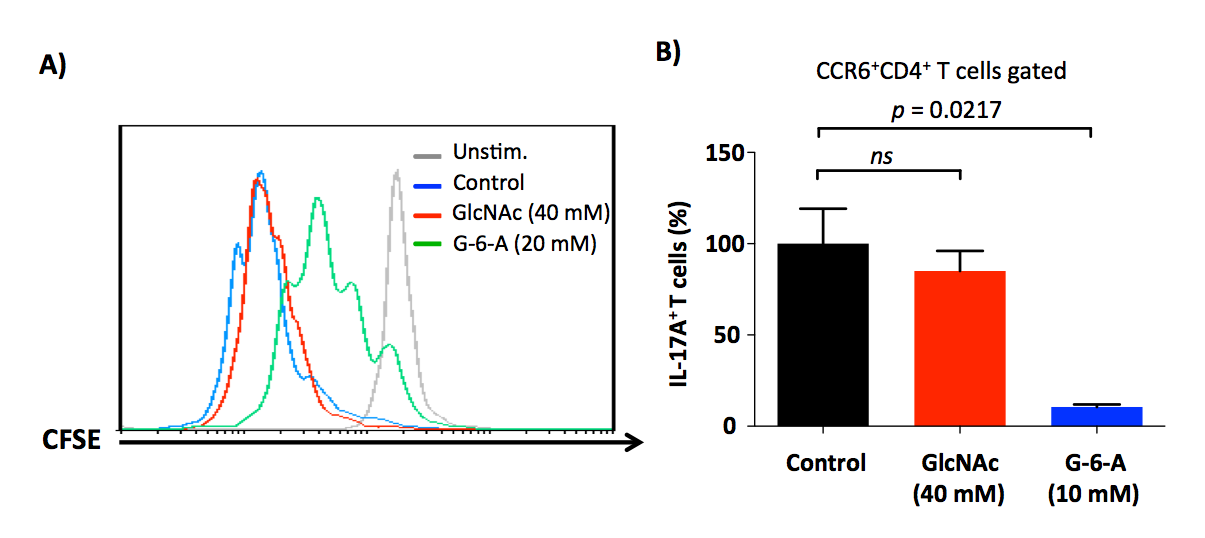

Supplement: S3 Fig — (A) Mouse splenic CD4+ T cells were labeled with CFSE, activated for 72 hrs with anti-CD3ε + anti-CD28, and treated with GlcNAc (40 mM) or G-6-A (20 mM). The experiment is representative from at least two independent experiments. (B) Human CCR6+CD4+ T cells were sorted and activated with anti-CD3ε + anti-CD28 under TH17-inducing conditions with control (untreated), GlcNAc (40 mM) or G-6-A (10 mM) for 5 days. The experiment is repeated twice with similar results. Error bars represent the means ± standard error of duplicate treatments. p values were determined by one-tailed t-test. (TIFF) [file pone.0214253.s003.tiff]

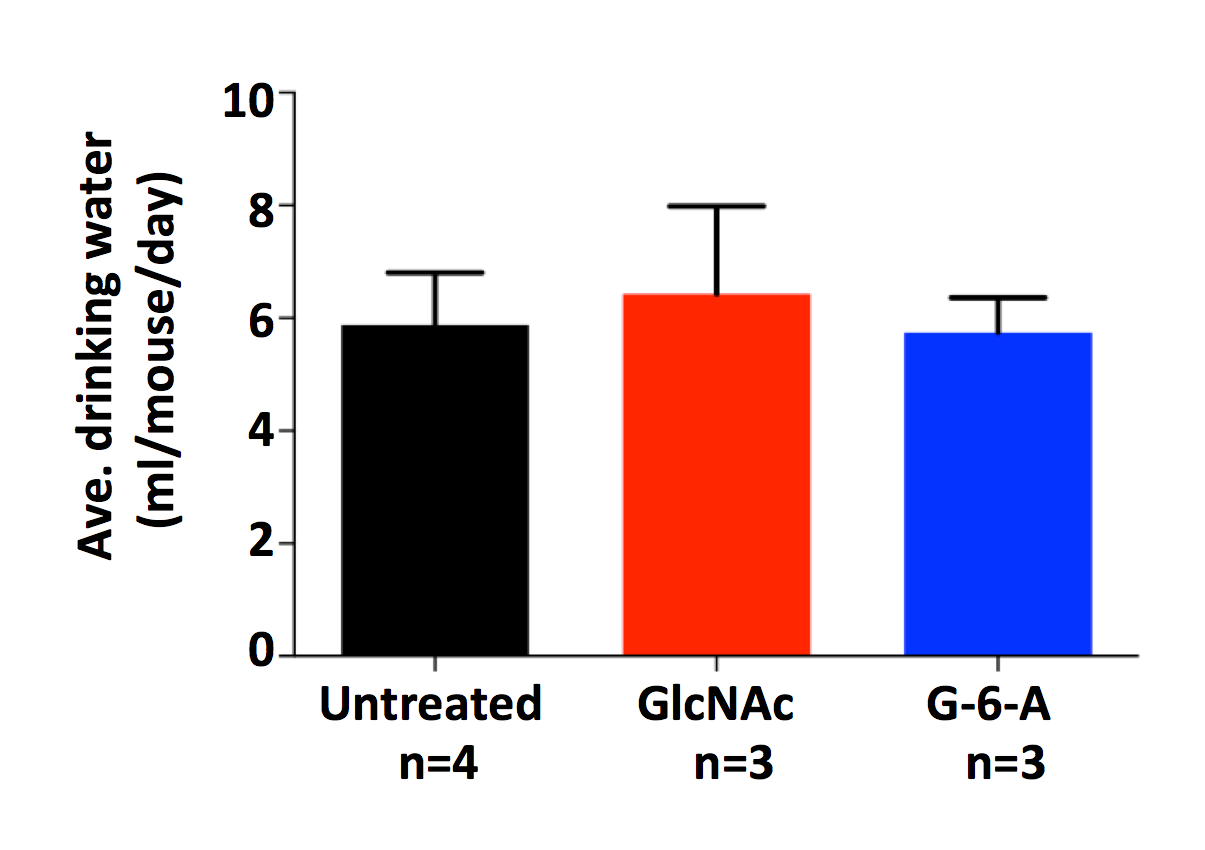

Supplement: S4 Fig — C57BL/6 Mgat5+/- mice were provided GlcNAc or GlcNAc-6-Acetate (G-6-A) at 0.25 mg/ml in their drinking water daily 5 days prior to MOG35-55 immunization and for 5 days post-immunization. Shown is the average amount of water consumed per mouse per day over the 10 day period of treatment. (TIFF) [file pone.0214253.s004.tiff]
